# Analysis of the clinical outcomes of microbial contamination caused by environmental contamination of the embryology laboratory during IVF-ET treatment cycles

**DOI:** 10.1186/s12884-023-05516-6

**Published:** 2023-03-18

**Authors:** Tingting Zheng, Qinhua Li, Ningjing Chen, Peiyao Du, Hong Ye

**Affiliations:** Department of Obstetrics and Gynecology, The First College of Clinical Medical Science, Yichang Central People’s Hospital, Three Gorges University, 183 Yiling St, Yichang, Hubei 443003 China

**Keywords:** Bacterial contamination, In vitro fertilization, Embryology laboratory, Pregnancy outcomes

## Abstract

**Background:**

Bacterial contamination may cause loss of or damage to cultured oocytes or embryos, resulting in the lack of transplantable embryos during IVF embryo culture. However, there are few reports about IVF embryo contamination caused by embryology laboratories. In this work, we evaluated clinical pregnancy outcomes and the risk of maternal and infant complications after embryo contamination caused by environmental pollution during IVF.

**Methods:**

The authors retrospectively analyzed 2490 IVF-ET ovulation induction therapy cycles in the Reproductive Center of Yichang Central People's Hospital from January 2015 to May 2022. According to the presence or absence of embryo culture medium contamination, the two groups were divided into an embryo contamination cycle and a nonembryo contamination cycle. The primary outcome parameters were the characteristics and progress of embryo culture medium contamination. Embryo laboratory outcomes, pregnancy outcomes, and maternal and infant complications were secondary outcome parameters.

**Results:**

One case of embryo contamination originated from semen contamination. The remaining 15 cases involved environmental contamination outbreaks in embryo culture chambers, caused by Staphylococcus pasteuri. Compared with conventional uncontaminated IVF cycles, the 15 cases of contaminated embryo cycles showed no significant difference in embryo laboratory outcomes, pregnancy outcomes, or maternal and infant complications except for a slightly higher rate of fetal growth retardation. Ultimately, 11 live-born infants were successfully delivered, of which 2 were premature. The remaining 4 patients did not become pregnant after 1–2 transfers due to a lack of transferable embryos.

**Conclusion:**

When the embryo culture medium is contaminated due to the environmental contamination of the IVF culture room, it is feasible to perform daily rapid rinsing of the culture medium and avoid blastocyst culture as remedial treatment. However, the long-term impact on offspring needs further prospective research.

## Introduction

Bacterial contamination may cause loss or damage to cultured oocytes or embryos, resulting in the lack of transplantable embryos during IVF embryo culture. Most studies have indicated that the bacterial contamination of IVF embryos is mainly caused by semen, and the positive rate of semen bacterial culture is 63%-100% [1]. Follicular fluid is the second most common contaminant [2], and the positive rate of bacterial culture is 9%-27% [3, 4]. There are few reports about IVF embryo contamination caused by embryology laboratories. Nevertheless, even the strictest laboratories are prone to contamination [5], as many reagents, devices, equipment, personnel, and even the environmental air represent a potential risk of contamination [6]. While there are numerous protocols and guidelines for IVF embryology laboratory practices that aim to reduce the possibility of introducing an adventitious agent into the embryology laboratory [7], there are no standard protocols available to detect and monitor sources of contamination by biological fluids and bacteria and fungi in the environmental air. The low number of publications and case reports dealing with the prevalence of microorganisms in ART laboratories suggests that the number of contamination events is largely underestimated. Among the 2490 IVF cycles included in this study, we retrospectively analyzed the clinical results and prevention measures in 15 patients with embryo contamination caused by the environmental contamination of an embryology laboratory with Staphylococcus pasteuri.

## Materials and methods

### Study setting and design

Among the 2490 IVF-ET cycles conducted at Yichang Central People's Hospital from January 2015 to May 2022, 1887 cycles were performed with conventional IVF, 366 cycles were performed with intracytoplasmic sperm injection (ICSI), and 237 cycles were performed with early rescue intracytoplasmic sperm injection (RICSI). In total, embryo cultures from 16 IVF-ET cycles were determined to be contaminated. One event occurred in 2019, where semen contamination was the source of embryo contamination. The remaining 15 cases of contamination all occurred in May 2020. After a comprehensive investigation, it was ultimately considered that the source of contamination was the accumulation of water in the interlayer between the ceiling and the top floor of the embryology laboratory, which caused Staphylococcus pastoris to reproduce and then contaminate the culture environment of the entire embryo culture room through the laminar flow purification system. In the contaminated embryo group, the average age of the women was 32.53 ± 4.31 years, the average age of the men was 35.73 ± 4.48 years, and the average infertility time was 3.33 ± 1.97 years. The causes of infertility included 8 cases of fallopian tube factors, 3 cases of ovarian dysfunction, 2 cases of ovulation disorders, and 2 cases of male oligoasthenospermia. ICSI was used in 3 cases, RICSI was used in 1 case, and IVF was used in the other 11 cases.

## Methods

According to the age of patients or ovarian reserve function, the 15 patients with embryo contamination were treated with the follicular phase long protocol (2 patients), antagonist protocol (10 patients), and PPOS protocol (3 patients) for ovarian hyperstimulation. The obtained oocytes were washed in K-SIGB medium (supplied by Cook, UK)to remove follicular fluid and blood and then put into a double-well culture dish containing K-SIFM medium (supplied by Cook, UK) in a 6% CO2 incubator at 37 ℃ for 6 h before insemination. Semen was obtained by masturbation after abstinence for 2–7 days. Semen quality was evaluated according to WHO standards after complete liquefaction and mixing. Semen was optimized by gradient centrifugation and the swim-up method. Conventional IVF was performed in mineral oil-covered microdroplets for short-term fertilization. After 4–6 h of sperm-egg incubation, the oocytes were removed from the sperm environment, the granulosa cells were removed, dipole exclusion was observed to decide whether to perform RICSI, and the oocytes were transferred to K-SICM medium (supplied by Cook, UK) for oil-covered microdroplet culture. The oocytes were observed under an inverted microscope on the day after insemination (18 h), and fertilization was considered normal when double protoplasts and dipoles were observed. After insemination, the cleavage of fertilized eggs was noted. High-quality embryos were defined as those with a normal cleavage timing, a uniform or approximately uniform blastomere size, an equal or approximately equal number of blastomeres, a fragmentation rate of 20% or less and a uniform texture. One or two high-quality Day 3 embryos or Day 5 blastocysts were selected for transfer, or all embryos were vitrified and thawed for transfer according to the clinical situation. Urine HCG or blood HCG was measured 14 days after embryo transfer. The pregnancy sac and fetal heartbeat were observed by vaginal color Doppler ultrasound 40 days after embryo transfer.

### Treatment of contamination

When the embryologist visually observed that the culture droplets were cloudy and filled with densely moving punctate or rod-shaped microorganisms under an inverted microscope, the inner diameter of the glass tube was drawn with an inner diameter of 120–140 μm, and the surface paraffin oil was carefully removed. The cells were blown repeatedly from the bottom of the microdrop dish to ensure that the colonies fell off the bottom of the dish. The contaminated culture droplets, blank culture droplets, follicular fluid, and semen collected in advance were cultured for the identification of bacteria. Embryos were removed from contaminated droplets and placed in organ-well culture dishes containing K-SIFM medium equilibrated at 37 ℃℃ and 6% CO_2_ with an oil overlay. After repeated washing, the embryos were transferred to new culture droplets for further culture [3, 8]. Then, the culture dish containing the embryos observed and media was replaced every 8 h until contamination was absent. On the third day, cleavage and contamination clearance were observed for transfer, embryo freezing, or blastocyst culture.

### Thorough disinfection of the embryology laboratory

Hypochlorite (0.5%) was used to disinfect the floor and instruments of the embryology laboratory. The disinfection of the air environment of the embryology laboratory mainly depends on the continuous purification laminar flow. Ultraviolet rays should be avoided because they can produce ozone and have adverse effects on embryos and culture reagents. Hydrogen peroxide (3%) is often used to wipe laboratory tables contaminated by blood and semen because it can interfere with the microbial enzyme system and affect metabolic activity. The components and consumables that can be disassembled from the incubator should be sterilized by a high temperature and damp heat as best as possible. Glass doors and inner liners should be cleaned with a special incubator cleaner, and glassware should be sterilized using a high temperature and dry heat. Awareness of contamination prevention among IVF embryology laboratory staff should be strengthened. Embryologists need to use disposable operating utensils and tools, maintain aseptic operations, and reasonably store embryo culture fluid.

### Statistical analysis

The percentages of each cycle and contaminated droplets and the outcomes of fertilization, cleavage, and blastocyst culture in the embryology laboratory are expressed as rates or constituent ratios. Clinical pregnancy, implantation, live birth, and adverse pregnancy outcomes after embryo transfer are expressed as constituent ratios. Comparisons were made using a chi-square test or Fisher's exact test. Statistical analysis of the data was performed using SPSS 19.0 software. A *p* value < 0.05 was considered statistically significant.

## Results

### Causes of contamination of the culture environment in the embryology laboratory during IVF-ET treatment cycles and its resulting characteristics of embryo culture fluid contamination

The first case of embryo contamination was initially identified in a 31-year-old woman whose D5 embryos fertilized by IVF were observed to have partial cloudy microdrop cultures and occasional spherical microorganisms. Subsequently, two other women had full or partial microdrop contamination in their D5 and D4 embryos, respectively, within 2 days, so contamination of the culture room environment was suspected. After a comprehensive inspection of the surfaces of the objects in each room, embryologists and related personnel, embryo culture fluid, incubators, petri dishes, laminar flow unit filters, ultraclean table filters, etc., and sampling for bacterial culture, it was ultimately determined that pond water had seeped into the culture room lampshade from a leaky penthouse through the hole of an electric wire; The seepage was caused by the continuous rainy season that occurred half a month before the first culture fluid contamination was identified, which contained the exact same bacterium as that in the contaminated culture fluid. The culture fluid contamination incident was considered to be related to the accumulation of water and bacterial multiplication caused by the leakage of water from the penthouse into the interlayer between the ceiling of the lower culture room and the penthouse during the rainy season.

**Fig. 1:**
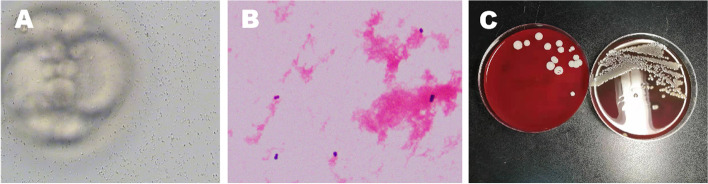
Contamination of embryos under a general inverted microscope. **A**: Contamination of embryo culture medium caused by the reproduction of Staphylococcus Pasteur. Microscope magnification is 200x. **B**: The centrifuged colony sediment smear was directly and microscopically examined as cocci after Gram staining. Microscope magnification is 200x. **C**: The colonies grew well after 48 h of inoculation on blood agar plate medium cultured in a 37-degree incubator set to 5% CO2

There were 15 IVF patients with contaminated embryos, with an incidence of 0.60% (15/2490). The time of contamination was concentrated within one month. Colonies were collected from embryo culture droplets in the 15 contaminated embryos, and the sediment smears were centrifuged to obtain cocci after Gram staining (Fig. 1). Then, the agar plate medium was cultured for 48 h, and the same bacterium—Staphylococcus pastoris—was identified by microbial mass spectrometry. However, no bacteria were detected in the follicular fluid or semen samples. Generally, bacterial colonies appeared 1–5 days after ovum retrieval. Two cases of contamination became apparent on the first day after ovum administration, 5 became apparent on the second day, 4 became apparent on the third day, 3 became apparent on the fourth day and 1 became apparent on the fifth day. After vitrification and thawing, affected embryos only need to be cultured for 8 h, and colonies can grow again. The incidence of embryo contamination on different culture days was found to be highest on Day 2 (33.33%), followed by Day 3 (26.67%), and lowest on Day 5 (6.67%). The droplet contamination rate of Day 2 samples was up to 8.09%, which was higher than that of other days, and the contamination rate of other days showed no significant difference (Table 1).

**Table 1:** Comparison of contamination of embryonic droplets caused by contamination of the culture environment on different culture days

Time of discovery of contamination	Contamination cycle number	Droplet contamination rate	X2	P
Day 1	13.33(2/15)	2.31(4/173)	11.280	0.024
Day 2	33.33(5/15)	8.09(14/169)^a^		
Day 3	26.67(4/15)	2.31(4/155)		
Day 4	20(3/15)	3.51(4/151)		
Day 5	6.67(1/15)	4.3(5/147)		

Percentages are preceded by parentheses. Droplet contamination rate = contamination droplets/examination droplets

^a^There was a significant difference between Day 2 and the other days and no significant difference among the other days

In the 15 cases of contaminated embryo culture dishes, the contamination was sporadic. The colonies did not spread to all of the microdroplets in the whole culture dish. The unused culture droplets were occasionally involved; there was no significant correlation between the occurrence of colonization and whether it was in culture droplets with embryos, culture droplets with embryo washes, and unused culture droplets. The contamination of embryo culture droplets progressed faster. If the culture medium was not changed, the colonies of contaminated drops could gradually be aggravated after 6–8 h of culture, while most uncontaminated drops had no obvious change, and a small number turned into contaminated drops after 1–2 days.

### Embryo development and pregnancy outcomes after embryo contamination caused by culture environment contamination during the IVF cycle

The fertilization, cleavage, high-quality embryo, transfer, pregnancy, and offspring follow-up rates of the 15 contaminated embryos are shown in Table 2. The 2PN rate of the 15 contaminated embryos was 74.58%, the 2PN cleavage rate was 99.10%, and the high-quality embryo formation rate was 68.18%. These were not significantly different from those of 63.33, 99.04, and 74.83%, respectively, in the conventional IVF cycle group (*P* > 0.05) (Table 3).

**Table 2:** Embryo culture and pregnancy outcomes of 15 patients with embryo contamination

No	1	2	3	4	5	6	7	8	9	10	11	12	13	14	15
Age of Female	31	27	35	31	30	27	42	31	31	42	28	38	28	38	29
Age of Male	32	37	35	41	29	35	44	34	31	52	29	37	32	37	31
Insemination mode	IVF	IVF	IVF	IVF	IVF, RI	ICSI	IVF	IVF	IVF	ICSI	IVF	IVF	ICSI	IVF	IVF
Oocytes retrieved	5	20	12	14	7	11	4	8	25	4	8	18	9	13	15
2PN fertilization	3	12	5	12	5	5	2	4	18	4	4	10	9	8	10
Abnormal fertilization	1	6	1	1	2	0	1	2	5	0	2	3	0	3	3
2PN cleaved	4	18	6	13	6	6	3	6	23	3	6	13	9	11	13
High-quality embryo	0	4	3	10	2	5	2	3	12	2	3	10	7	3	9
Day of contamination	5	5	4	4	4	1	1	2	2	5	3	3	3	3	2
Total embryo contamination cycle	N	Y	N	N	N	N	N	N	N	N	N	N	N	N	N
Available embryos	1	8	3	10	5	4	2	5	16	2	4	6	6	5	10
Fresh embryo transfer	Y	N	Y	Y	Y	Y	N	Y	N	N	Y	N	Y	N	N
First embryo transfer number	1	1	2	1	2	1	2	1	1	2	1	1	1	1	1
Embryo implantation	0	1	0	1	2	1	0	1	1	0	1	1	0	0	1
Adverse pregnancy outcome	-	-	-	-	-	-	-	EP	-	-	-	-	-	-	SAB
Second embryo transfer number	-	-	1	-	-	-	-	1	-	-	-	-	1	2	1
Embryo implantation	-	-	0	-	-	-	-	1	-	-	-	-	1	1	1
Live birth	-	1	-	1	2	1	-	1	1	-	1	1	1	1	1
Gestational week	-	39 + 3	-	37	35 + 5	36 + 4	-	37 + 3	38	-	39 + 1	-	38	37 + 5	37
Low birth weight					Y	Y									
Birth weight(kg)	-	3.0	-	2.9	2.0/1.8	2.5	-	2.7	4.1	-	3.6	2.7	3.2	3.4	2.8
Sex	-	F	-	F	M/M	M	-	F	F	-	M	M	M	F	F
Neonatal asphyxia	-	N	-	N	Y	N	-	N	N	-	N	N	N	N	N
Intrauterine growth retardation	-	N	-	N	Y	N	-	N	N	-	N	N	N	N	N
Fetal distress	-	N	-	N	Y	N	-	N	N	-	N	N	N	N	N
Premature rupture of membranes		Y	-	N	N	N		N	N		N	N	N	N	Y
Birth defect rate	-	N	-	N	N	N	-	N	N	-	N	N	N	N	N
1 year after birth	-	norm	-	norm	norm	norm	-	norm	norm	-	norm	norm	norm	norm	norm

*EP* Ectopic pregnancy, *SAB* Spontaneous abortion, *F* Female, *M* Male, *norm* Normal, *Y* Yes, *N* No

**Table 3:** Effects of embryo contamination on embryo development (%)

	**Embryo contamination cycle**	**Embryo contamination-free cycle(Regular IVF Cycle)**	**X2**	**P**
Cycle	15	1887	-	-
2PN fertilization rate	74.58(111/173)	63.33 (13,064/20628)	0.051	0.821
2PN cleavage rate	99.10 (/110/111)	99.04 (12,938/13064)	0.005	0.946
High-quality embryo rate	68.18 (75/110)	74.83 (9681/12938)	2.553	0.110
Blastocyst formation rate	67.54 (77/114)	62.79 (7623/12140)	1.092	0.296
High-quality blastocyst rate	40.35 (46/114)	39.21 (4760/12140)	0.062	0.804
Clinical pregnancy rate	62.50 (5/8)	52.19 (1182/2265)	2.816	0.133
Implantation rate	60.00 (6/10)	39.52 (1457/3687)	1.750	0.208
Spontaneous abortion rate	20.00 (1/5)	13.11 (155/1182)	0.207	0.506
Neonatal asphyxia	0(0/12)	0.61(6/988)	0.073	1.000
Neonatal pneumonia	0(0/12)	0.30(3/988)	0.037	1.000
Intrauterine growth retardation rate	9.09(1/12)	9.92(98/988)	8.762	0.003
Fetal distress	9.09(1/12)	7.49(74/988)	0.012	0.610
Amniotic fluid contamination rate	0(0/12)	0.51(5/988)	0.061	1.000
Prematurity rate	9.09(1/12)	15.690(155/988)	0.487	0.704
Low birth weight rate	18.18(2/12)	8.40(83/988)	1.042	0.272
Premature rupture of membranes rate	18.18(2/12)	13.16(130/988)	0.127	0.665
Birth defect rate	0(0/12)	1.52(15/988)	0.185	1.000

Percentages are preceded by parentheses. Differences between contaminated embryos and uncontaminated embryos were compared using Pearson's chi-square test or Fisher's exact test (*P* < 0.05)

To avoid the patients being forced to give up embryo transfer, after receiving the informed consent of the patients, the authors performed fresh embryo transfer of contaminated embryos in 8 patients in the embryo contamination group, 5 of whom became pregnant and delivered. Patient 8 underwent laparoscopic tubal fenestration for embryo retrieval due to ectopic pregnancy, and Patient 15 underwent genetic testing due to spontaneous abortion villi in the second month of pregnancy, suggesting trisomy 16. Both patients had embryos transplanted during a freeze‒thaw cycle and became pregnant and delivered. In Patients 2, 9, and 12, embryo transfer was cancelled to prevent the occurrence of ovarian hyperstimulation syndrome. In Patients 7, 10, 14, and 15, embryo transfer was cancelled due to the high *P* value, and the embryos were all vitrified. Then, 1–2 embryos were thawed and transferred, leading to pregnancy and delivery. Patients 1, 3, 7, and 10 had no remaining embryos after 1–2 transplants that did not result in pregnancy.

There was no significant difference between the embryo contamination group and the conventional IVF cycle group in terms of neonatal distress, amniotic fluid contamination, neonatal asphyxia, premature infants, low birth weight, the incidence of premature rupture of membranes, or birth defects (*P* > 0.05). However, the rate of intrauterine growth retardation in the embryo contamination group was lower than that in the conventional IVF cycle group (*P* < 0.05). The newborns were followed up to one year after birth, and there was no abnormal growth, development, or intelligence of the delivered neonates during the follow-up.

## Discussion

### Causes and characteristics of environmental contamination in the embryology laboratory

The IVF embryo culture environment includes the internal environment of the IVF embryology laboratory, laminar flow systems, various instruments and equipment, incubators, culture mediums, culture dishes, various glass tubes and plastic tubes as well as operators [9, 10]. All reproductive medicine centers are aware of the importance of the embryo culture room environment, so resulting contamination is rare [11]. However, once contamination of the cultivation environment occurs, the contamination spreads rapidly. Cottell [8] cultured semen samples before and after treatment and detected a small amount of Staphylococcus epidermidis and Streptococcus viridis in the treated semen, while there were no such bacteria in the untreated original semen. This suggested that contamination by these two bacteria may result from the cultivation and operation process in the embryology laboratory [1]. In this study, the first three patients had embryos contaminated by the same bacteria within two days, which drew the attention of the embryologists. It was considered that the contamination of these embryos may have come from the culture environment. According to the operation procedures, quality control, and laboratory management strategies in the laboratory guidelines for human embryology and andrology (revised version 2008) [12] and the IVF laboratory operation guidelines of the European Association of Human Reproduction and Embryology [13], the culture medium, incubator, culture dish, laminar flow unit filter, and ultraclean table filter were quickly checked and sampled for bacterial culture. Finally, we consider that the source of the contamination was the repeated rain in the plum rain season, which led to the leakage of water from the top floor into the interlayer between the ceiling and the top floor of the culture room, resulting in water accumulation and bacterial reproduction. After the pond was treated and pasteurized, a pipe was installed above the interlayer to drain water. All skylights of the interlayer between the floors were opened for ventilation, and the patients were informed to stop IVF ovulation induction, egg retrieval preparation, and the transplantation cycle. After 2 weeks, all embryology laboratory operations were terminated, the ceiling of the culture room was fully disinfected, and the ground was waterproofed. Therefore, the IVF culture system and environment can also be potential sources of embryo contamination, which should be considered. In this study, we found that when the roof leaked and bacterial colonization caused contamination of the culture room environment, the sterilization effect was likely to be ineffective, even after replacing the type, model, and batch of the culture medium, culture dish, and incubator. It was still necessary to close the IVF embryology laboratory for comprehensive disinfection and sterilization of the ceiling and waterproofing treatment of the ground affected by the water leak. Although the embryology laboratory will be established on the highest floor, which is conducive to air circulation and air quality improvement, the construction of the waterproofed roof floor needs special attention regarding its back surface, and the water surface should be developed with high-quality waterproofing materials and receive regular maintenance to prevent years of roof water seepage.

### Differences in embryo culture fluid contamination caused by environmental contamination in the embryology laboratory compared with semen and follicular fluid contamination

We found that on the first to fifth days of embryo culture in vitro, the discovery rate of Day 2 contamination was the highest, followed by Day 3 and Day 4. It is speculated that this may be related to the source of microorganisms, growth mode, and half-life of antibiotics in the culture medium [14]. However, different from the high contamination rate of embryo culture medium caused by semen or follicular fluid, the contamination rate of embryo culture medium caused by environmental contamination, which is sporadic, is only 8.09% (Day 2). This is considered to be related to the low concentration of bacteria in the environment due to the continuous operation of the laminar flow purification system [10].

Different from the individual sporadic culture fluid contamination caused by semen and follicular pollution, the culture fluid contamination caused by environmental pollution develops and spreads very rapidly. In this study, due to continuous rain in the rainy season, the interlayer between the top floor and the ceiling of the culture room caused water accumulation, which led to Staphylococcus Pasteurella reproduction [15]. Staphylococcus pasteuri was detected in the water in the interlayer, the light fixture with the slit on the ceiling, and the embryo culture medium. Staphylococcus pasteuri is widespread in drinking water treatment plants [16, 17]. The Department of Biotechnology reported that the use of diatomite-immobilized hydrolase from Staphylococcus pasteuri can pretreat coconut factory wastewater and its effect on anaerobic digestion [18, 19]. The results of studies indicate that the bacteria causing culture fluid contamination are mainly semen-derived E. coli and fungi [20]. E. Coli contaminated embryos to a significant degree and other bacteria contaminated embryos to a lesser degree. However, in this report, embryo contamination caused by the reproduction of Staphylococcus pasteuri due to rainwater deposition does not seem to have a significant effect on egg fertilization, cleavage, high-quality embryos, transplantation, pregnancy, or fetal malformation in the short term. This may be related to the bacteria's strong ability to degrade organic matter and significantly improve anaerobic digestion. This conclusion is consistent with the opinion that system-positive ET catheter culture in asymptomatic women does not increase the risk of IVF failure [21]. Positive cultures may not be associated with poor IVF outcomes, but a large-sample statistical analysis is still needed.

### Prevention and treatment of embryo contamination: Remedial measures for embryo contamination caused by culture environment contamination during IVF cycles

The treatment of contaminated embryos involves the repeated washing of embryos. At present, the culture medium used in the ART embryology laboratory contains antibiotics, and most bacteria can be removed by gradient centrifugation and swim-up methods. Cottell [3] reported that 95%-100% of bacteria can be removed by treating semen with a culture medium containing antibiotics by the upstream method, suggesting that antibiotics play a key role in inhibiting the growth of bacteria in the culture medium [22]. In this study, 0.60% of the embryos were contaminated by bacteria despite the presence of antibiotics, such as gentamicin, in the sperm handling, egg culture, embryo culture and washing replacement media (supplied by Cook, UK). This may be due to the antibiotic resistance of bacteria, which may lead to a large number of bacteria in embryo culture droplets and cause serious damage to the development of embryos. Therefore, more effective measures are needed to prevent embryo contamination.

In this study, the method of repeated washing of culture medium twice a day can basically ensure that microdroplets and embryos will not be contaminated again within 4–6 h. After continuous washing for 1–2 days, most of the embryos were not contaminated again in the process of continuing to culture blastocysts. Only 4 microdroplets from 2 patients were contaminated again with culture medium. For every 2–3 contaminated culture droplets washed, the glass tube was replaced with a new tube. Then, the cleaned glass tube was used to absorb new culture medium to clean the embryos. This study suggests that fluid replacement for contaminated embryos may improve the developmental outcomes of contaminated embryos because culture fluid replacement removes most bacteria, and the newly added antibiotics effectively inhibit bacterial growth so that the surviving embryos can continue to develop.

Reduce the number of blastocysts. There are no data to confirm whether contaminated embryos should be transferred after treatment. To avoid the patients being forced to give up the cycle transfer, after good communication with the patients, the authors treated the contaminated embryos and performed embryo transfer to obtain clinical pregnancy, which achieved good results. The remaining high-quality transplantable embryos were vitrified. After thawing for 24 h, no recontamination was identified, so the embryos were transferred. In this report, there were 7 cases of thawing transfers, including 4 cases of pregnancy. Therefore, when embryo contamination is identified, it is suggested that transplantable embryos should be transferred or frozen on the third day, and blastocysts should continue to be cultured only for inferior embryos.

### ICSI insemination and semen optimization are ineffective against embryo contamination caused by environmental contamination

ICSI avoids contact between oocytes and semen, thereby reducing the possibility of semen contamination of embryos [20]. Additionally, this may be due to the consideration of PVP by single motile sperm during ICSI. In conventional IVF and ICSI, sperm only go through gradient centrifugation or upstream selection, most bacteria are removed, and the possibility of contamination is further reduced during a single sperm injection. On the other hand, this can reduce the risk of contamination. In the process of ICSI, oocytes are digested by enzymes and washed repeatedly. Seidman [23] reported that one patient's embryo was contaminated by bacteria three times and the patient became pregnant after using epididymis sperm for ICSI, suggesting that ICSI can effectively prevent embryo contamination caused by semen and follicular fluid. In this study, among the 15 patients with embryo contamination caused by the culture environment, 3 were inseminated by ICSI because of oligoasthenospermia on the male side. Finally, individual droplet embryo contamination still occurred on Days 1, 3, and 5. It is suggested that ICSI and gradient centrifugation plus upstream sperm selection cannot prevent embryo contamination caused by the environment.

## Conclusions

In this paper, we report for the first time that embryo contamination caused by Staphylococcus pasteuri colonization does not seem to have a significant effect on laboratory outcome and pregnancy outcome of embryos in the short term, suggesting that the outcome of embryo contamination is related to the type of bacteria contaminating the embryos. This study showed that when the environmental contamination of the IVF culture room leads to contamination of the embryo culture medium, it is feasible to take measures such as strengthening the daily rapid flushing of the culture medium and reducing blastocyst culture as remedial treatment. In addition, there are other methods, such as the removal of the zona pellucida of contaminated frozen blastocysts [24]. However, the present study has some limitations. First, microbial contamination of embryos can be detected only when the microorganisms grow to a certain amount, and it is difficult to detect microorganisms that grow slowly and multiply in small amounts without sufficient attention to ordinary inverted microscopy. Secondly, the sample size of this study was small, so the results may not be robust. However, considering the relatively rare incidence of culture fluid contamination due to environmental pollution and the interpretability of the results of this study, they are still presented. The reliability of the results needs to be corroborated by further studies. Finally, the long-term effects of embryo contamination on offspring require further prospective studies. Reproductive medicine clinicians and embryology laboratory personnel still need to pay attention to embryo contamination.

## Data Availability

The datasets used and analyzed during the current study available from the corresponding author on reasonable request.
